# Effect of water quality on ice hardness and skate-to-ice friction in ice rinks

**DOI:** 10.1007/s12283-025-00538-z

**Published:** 2026-01-29

**Authors:** Ryan H. S. Hutchins, Jiani Wang, Stefania Impellizzeri

**Affiliations:** Department of Chemistry and Biology, Toronto Metropolitan University, 350 Victoria Street, Toronto, M5B 2K3 Ontario Canada

**Keywords:** Ice hardness, Skate-to-Ice Static friction, Water treatment, Total dissolved solids (TDS), Non-destructive ice testing

## Abstract

Ice hardness and friction influence performance and safety in ice sports, yet the role of water quality remains poorly understood. This study examines how total dissolved solids in rink water affect ice characteristics using nondestructive hardness testing and a skate-to-ice static friction index measured under controlled rink conditions. Results show that temperature influences ice hardness and static friction, with colder ice being harder and exhibiting higher static friction. Water quality also plays a role, as lower total dissolved solid levels reduce static friction, with effects comparable to a 1$^{\circ }$C increase in ice temperature. However, ultra-pure water produced softer ice, while moderate total dissolved solid levels maintained ice hardness with minor static friction increases. These findings support hockey rink recommendations to maintain total dissolved solids near 80 to 100 ppm to balance ice hardness with friction performance. However, other sports, such as curling, may benefit from lower total dissolved solids for reduced static friction. Understanding these relationships informs best practices in rink maintenance and water treatment.

**Supplementary Information:**

The online version contains supplementary material available at 10.1007/s12283-025-00538-z.

## Introduction

Ice quality influences performance and safety in ice-dependent sports such as hockey, figure skating, speed skating, and curling. While ice quality encompasses multiple physical and environmental characteristics, two key factors influencing athlete performance are ice hardness [1] and friction at the ice surface [2]. Ice hardness influences contact mechanics and surface response. For skating, higher hardness is associated with brittle behaviour and reduced blade penetration, whereas lower hardness increases penetration and resistance due to plowing. In curling, surface hardness affects the deformation and plowing of ice asperities (microscopic surface features), influencing stone–ice friction and surface durability. Friction governs resistance to motion in ice sports, influencing skating speed and puck travel in hockey, while ice–stone friction controls draw weight, curl, and trajectory in curling. Higher friction increases resistance to motion and can reduce movement efficiency and technical precision. Maintaining these properties requires control over various environmental factors and the quality of the water used to create the ice [3]. While temperature and humidity have been widely studied and are closely monitored in arena operations, the influence of water quality on these performance-related properties has received less attention.

Water quality in this context is often described in terms of total dissolved solids (TDS), a measure of the combined concentration of inorganic salts (such as calcium, magnesium, and sodium), bicarbonate, and small amounts of organic matter. While moderate TDS levels are generally beneficial for drinking water, high concentrations (over 1000 ppm) can lead to taste issues [4]. The requirements for ice-making, however, are distinct: TDS may influence ice properties by altering freezing behaviour, modifying crystal structure, and affecting the mechanical characteristics of the ice itself [5, 6]. TDS is also correlated with water conductivity [4] and can influence pH and carbonate equilibria. Although industry guidelines increasingly reference TDS thresholds, these are based largely on operational experience rather than controlled experimentation. For instance, the International Ice Hockey Federation (IIHF) [7] recommends maintaining TDS levels below 120 ppm, while many National Hockey League (NHL) ice-makers prefer a narrower range of 80–100 ppm for desirable ice conditions [8]. Curling organizations such as World Curling [9] endorse the use of reverse osmosis water, which typically contains less than 10 ppm TDS. Despite these varied recommendations, few studies have tested how TDS and water chemistry influence the mechanical and surface properties of ice under realistic rink conditions.

Hardness is an influential property of ice that affects its ability to resist deformation and withstand the physical impacts experienced during sporting events. One of the earliest studies to examine ice hardness in the context of winter sports was conducted by Poirier et al. [1]. They measured hardness by dropping steel balls ranging from 8 to 540 grams from heights between 0.3 and 1.2 m (0.02 to 3 J impact energy) and analyzing the diameter of the resulting impact craters. Although destructive, this approach provided a foundational understanding of ice hardness and its relationship to environmental factors such as ice surface temperature and air humidity. Their results showed that ice hardness decreased with increasing ice temperature, following a linear trend, while the effect of air humidity remained inconclusive. The study was conducted in ice sports venues, ensuring that the measurements reflected real-world conditions. These findings emphasized the role of temperature in determining ice hardness and highlighted the need for systematic testing methods to evaluate ice properties under real-world conditions.

Research has since shifted toward developing nondestructive testing methods to assess ice hardness without compromising rink surfaces. Nondestructive testing techniques allow repeated measurements in situ, making them particularly valuable for monitoring ice quality in arenas. Recent studies, such as two Zhang et al. articles [5, 6], have applied indentation-based and rebound hardness tests to investigate the influence of water quality and TDS levels on ice strength and flexibility. However, the precise relationships between TDS concentration, ice hardness, and friction remain uncertain, necessitating further controlled experimentation.

In this study, we used two nondestructive methods to quantify ice hardness: portable rebound hardness tester (Leeb C and Leeb D) and indentation hardness tester (Shore D). To assess friction, we employed a custom-built apparatus designed to simulate a skater on skate blades, which allowed us to measure a static friction index between skate and ice surfaces. These tests, conducted on ice made with varying TDS levels, provide insights into how water quality can be tuned to enhance ice performance in arenas. All tests were performed on a scaled-down experimental rink equipped with high-resolution temperature probes and complete climate control, ensuring precise environmental regulation.

## Methods

**Fig. 1:**
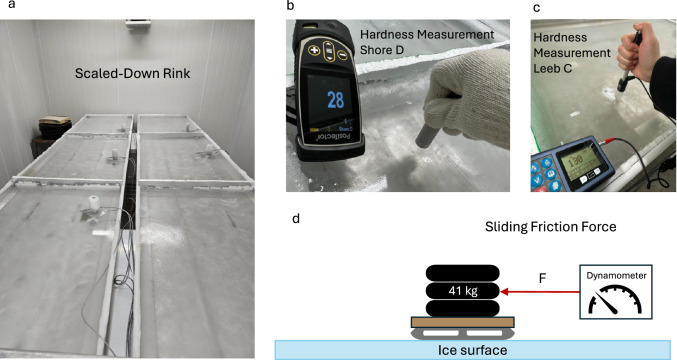
Experimental setup for ice hardness and friction testing: **a** Scaled-down experimental rink with six sections, each equipped with temperature probes to ensure controlled environmental conditions; **b** Shore D Durometer used for measuring indentation hardness; **c** Leeb C hardness tester, which measures rebound-based hardness; **d** Graphical representation of the experimental skater apparatus for friction testing, consisting of a 41 kg load applied to two hockey skate blades and connected to a dynamometer to measure the sliding force

### Rink apparatus and experimental design

The scaled-down experimental ice rink (Fig. 1a) in our laboratory was divided into six sections, each measuring 60 $\times$ 95 cm, with a temperature probe tower positioned in each section. Each tower contained three 10K Type 2 thermistors (Greystone Energy Systems, Moncton, Canada, ±0.03$^{\circ }$C accuracy) embedded at distinct depths within the ice: one at the bottom resting on the floor beneath the ice, one at the mid-depth (15 mm), and one frozen into the ice surface at 32 mm. The surface probe was used for all reported ice surface temperature measurements.

Two independent experiments were conducted. In each, ice was built fresh using three randomly assigned sections built with untreated Toronto tap water (220 ppm TDS, used as the control) and the other three sections built with one of the treatment waters (described in Section 2.2; either 80 ppm or 5 ppm TDS). This allowed each treatment to be compared against the same control under identical environmental conditions, with replication across experiments to strengthen statistical inference.

Each section was randomly assigned a water type during the experiments to ensure robust and unbiased comparisons. The ice was built by freezing a thin layer of water at a time, just enough to cover the surface completely, allowing for uniform ice formation across sections. About 400 mL of water was added to each section per freezing cycle, forming layers of  0.7 mm thickness. Ice thickness was maintained at 32 mm, following industry standards set by the IIHF [7] to replicate real-world rink conditions. Thickness was monitored using visual depth markers appropriate to the reduced spatial scale of the experimental rink. The air temperature and humidity were maintained between 9–11$^{\circ }$C and 40–50%, in accordance with IIHF guidelines. These conditions help minimize condensation on the ice surface while preventing excessive sublimation, both of which can negatively impact ice performance.

The scaling of the experimental rink was designed to approximate conditions found in full-scale arenas. An NHL-sized ice surface measures 1,516.8 m$^{2}$, while an international ice surface measures 1,764 m$^{2}$, compared to the 3.53 m$^{2}$ scaled-down rink used in this study. Refrigeration was provided by two Glycol Deck 250 (Vin service, Zanica, Italy) chillers, with a combined cooling capacity of 1.17 kW. A study of 12 community ice rinks in Quebec reported cooling capacities ranging from 247 kW to 310 kW [10]. Whereas, the IIHF recommends 300 to 400 kW of cooling capacity for international competition [7]. When normalized by surface area, the cooling capacity of the scaled-down rink was 332 W/m$^{2}$, which is higher than the 162 to 204 W/m$^{2}$ reported for full-scale community rinks and the 170 to 227 W/m$^{2}$ recommended for international competition. This increased cooling capacity per unit area helps maintain a stable ice surface despite the higher heat transfer rates expected in a small-scale setup.

To assess the effects of temperature and water treatment, friction and hardness measurements were taken at surface temperatures ranging from -2$^{\circ }$C to -7$^{\circ }$C. For each treatment in each experiment, 24 measurements were recorded across this temperature gradient, allowing for statistical comparisons between water types and thermal conditions. To ensure surface consistency across temperatures, the uppermost layer of ice was melted using a heat gun between measurement sets. This process created a fresh, uniform surface for both hardness and skate-on-ice static friction tests, minimizing the effects of prior tool contact or accumulated frost.

### Water quality and treatment

The water used to construct the ice consisted of three types. Untreated Toronto tap water was used as a baseline, representing the local municipal supply at about 220 ppm TDS [11]. Additionally, a Jet Ice Pro-Ice Mach 2 Reverse Osmosis system was used to produce treated water at levels of 80 ppm and 5 ppm. Reverse osmosis (RO) removes dissolved inorganic ions and organic compounds (including dissolved organic carbon) by applying pressure to force water through a semipermeable membrane, effectively lowering both ionic content and conductivity [12]. The IIHF [7] guidelines classify water with TDS <80 ppm as excellent and set 120 ppm as the minimum quality cutoff for ice making. Similarly, many NHL ice-makers maintain TDS between 80–100 ppm for desirable ice quality [8]. Meanwhile, World Curling [9] recommends the use of RO water, which typically results in TDS values between 0 to 5 ppm. The selected TDS levels cover a full range of acceptable and unacceptable water quality standards, with 220 ppm representing less-desirable water, 80 ppm falling within the ideal range for hockey, and 5 ppm serving as an ultra-purified condition for comparison.

Water chemistry was controlled and monitored to ensure consistent experimental conditions. TDS levels were verified using ion chromatography (Metrohm Professional Vario, Herisau, Switzerland); typical repeatability was $<{2}\%$ for major dissolved ions. Bicarbonate and carbonate ions were quantified using a Total Organic Carbon analyzer with a nitrogen module (Analytik Jena Multi N/C 3100, Jena, Germany), with an accuracy of ±5%. pH and electrical conductivity were measured using a SevenExcellence meter (Mettler Toledo, Greifensee, Switzerland); pH measurements were calibrated with a resolution of 0.001 pH units (accuracy ±0.002), and conductivity measurements had an accuracy of ±0.5%. Organic carbon and nitrogen were included in TDS calculations to provide a comprehensive assessment of water composition. Water “hardness” is defined here as the sum of calcium and magnesium ions and is distinct from the physical hardness of ice discussed elsewhere in this study. Replicate analytical measurements showed coefficients of variation below 1% across reported water chemistry parameters. Because TDS was calculated as the sum of measured constituents, uncertainty in TDS reflects cumulative analytical uncertainty across ions; TDS is therefore reported as mean ±5% error.

### Hardness testing

Ice hardness was measured using nondestructive testing methods: portable rebound hardness testers (Leeb C and Leeb D; DHT-100, TIME Group Inc, Beijing, China) and an indentation hardness tester (Shore D; PosiTector with SHD-D probe, DeFelsko Corporation, Ogdensburg, USA) (Fig. 1b, c). Each measurement was the mean of eight tests conducted in each section of the rink. Before ice testing, each hardness device was evaluated on its calibration standard. The Shore D tester had a coefficient of variation (CV) of 2.8%, while the Leeb C and Leeb D testers had CVs of 0.41% and 0.50%, respectively, across eight repeated measurements. These methods are considered nondestructive because they allow for in situ hardness testing with minimal impact on the ice surface. While the Shore D and Leeb hardness testers leave small indentations, the effect is less than the gouging and compression caused by skate blades during normal use. In contrast, destructive methods such as universal testing machines require ice samples to be removed from the rink and crushed. By allowing repeatable on-ice measurements without compromising the surface, these nondestructive techniques provide practical alternatives for assessing ice quality in operational rinks.

To avoid melting the ice during measurements, all hardness testers were placed on the aluminum plate adjacent to the rink for at least 30 min to thermally equilibrate. For the Shore D durometer, gloves were also worn during use to minimize heat transfer from the hand. These precautions helped reduce the risk of localized melting at the point of contact. A similar approach was taken by Zhang et al. [6], who used a low-temperature chamber to precondition their instruments prior to hardness testing.

The Leeb C and D testers differ in their impact energy, with the Type C tester delivering a lower impact energy of 3 mJ, compared to 11.5 mJ for the Type D tester. This difference in energy affects their sensitivity to surface conditions and material properties, with Type C generally suited for softer materials and thinner layers. Measurements were taken across a range of ice surface temperatures − 2$^{\circ }$C to − 7$^{\circ }$C) to simulate real-world variations encountered in professional ice arenas. According to IIHF [7] guidelines, ice surface temperatures between −4.5$^{\circ }$C and −5.5$^{\circ }$C are considered excellent, while −5.6$^{\circ }$C to −6$^{\circ }$C is rated as good, and temperatures below −6.5$^{\circ }$C are classified as the minimum acceptable level for playability. The temperature range used in this study was designed to capture both desirable conditions and less favourable temperatures that may occur due to fluctuations in refrigeration efficiency or arena-specific constraints. Testing at −2$^{\circ }$C provides insight into warmer, less ideal conditions, while extending to -7$^{\circ }$C allows for assessment at the lower end of the IIHF’s minimum quality threshold. This range ensured that the effects of temperature on ice hardness and friction were evaluated under conditions relevant to professional rinks.

### Friction testing

To assess friction, we employed a custom-built apparatus designed to simulate a skater on skate blades, which allowed us to measure a static friction index between skate and ice surfaces (Fig. 1d). The apparatus was fitted with two standard hockey skate blades from size 5.5D skates (26 cm length, 2.85 mm width), spaced 23 cm apart to replicate a realistic skating stance. Although we did not directly measure the roughness of the skate blades used in the tests, Lever et al. [13] reported typical hockey skate runner roughness values of 0.84 ± 0.55 µm, which provided a reasonable benchmark for comparison. The loaded apparatus had a total mass of 41 kg. This mass struck a balance between portability and replicating realistic skate pressures from lightweight skaters, such as youth hockey players or figure skaters. The coefficient of static friction ($\mu _s$) was calculated using the formula:

$$\begin{aligned} \mu _s = \frac{F }{ g \times m} \end{aligned}$$

where *F* is the measured force, *m* is the mass of the slider, and *g* is gravitational acceleration (9.8 m/s²). Force was measured with a digital force gauge (Precision Pro FC20; Torbal, Bohemia, USA; resolution 0.005 N; accuracy ±0.1%, manufacturer specifications). The device was operated in peak mode, which logs the peak force immediately prior to motion and corresponds to the static friction force. Each observation was calculated as the mean of eight replicate measurements per section. Within these sets of eight replicate measurements, the coefficient of variation was 3.7%. The apparatus was placed on the ice 30 min before testing to thermally equilibrate, and each measurement was preceded by a 15 s rest to allow the system to settle and replicate realistic pause conditions.

This test represents an index of the friction state under controlled conditions and is not meant to characterize all aspects of tribological behaviour of the ice surfaces built with different water treatments. Here, $\mu _s$ serves as an index because it captures the peak resistance before motion begins and reflects adhesion and deformation at the skate–ice interface. A rink example of static (rather than kinetic) friction is the initial motion of a curling stone at the start of a peel. While $\mu _s$ is the maximum resistance before sliding begins and kinetic friction (coefficient of kinetic friction, $\mu _k$) refers to the resistance during sliding, both are governed by the same interfacial mechanisms, primarily adhesion and plastic deformation across the real area of contact. As a result, the two forms of friction typically co-vary across ice temperature conditions [14]. To quantify this relationship, we digitized published static and kinetic friction data from ref. [14] (Fig. S1) and fitted a log-linear model relating $\mu _s$ to $\log (\mu _k)$, which explained most of the variance (Fig. S2, $R^2 = 0.986$). The digitized data and fitted relationship are provided in the Supplementary Information. Although not identical, this supports the use of static friction as a practical proxy for assessing changes in ice surface conditions.

Our use of a 41 kg mass is consistent with the theoretical basis of friction. According to the first rule of classical tribology [15], the coefficient of friction should be independent of the applied normal force. This principle has been validated in steel-on-ice systems by Calabrese et al. [16], who showed that friction coefficients remained stable across normal forces ranging from 100 N to 900 N, including values below and above the 402 N force used in our setup. Furthermore, a review by Kietzig et al. [17] noted that, while friction generally decreases with increasing normal force in ice friction systems, for steel sliders close to the freezing point the friction coefficient becomes independent of normal force. Accordingly, we consider our test apparatus and loading conditions suitable for generating a repeatable static friction index under rink conditions.

### Statistical analysis

All analyses were conducted in R statistical programming version 4.2.1 [18]. Plots were made with the *ggplot2* package [19]. We used mixed-effect models with the *lme4* package [20] to evaluate how ice hardness and friction respond to water treatment and surface temperature. This modeling approach is conceptually similar to standard linear regression but is specifically designed to handle grouped or hierarchical data, in this case, repeated measurements from the same rink sections.

In each model, surface temperature was treated as a continuous fixed effect, while water treatment (220 ppm, 80 ppm, 5 ppm) was modeled as a categorical fixed effect representing baseline shifts for each treatment. Rink section was included as a random effect to account for variability between experimental areas. Though termed “random”, these effects represent structured, repeatable differences between sections (e.g., minor airflow or freezing variations) and are modeled as separate intercepts, allowing each section to have its own baseline. This prevents underestimating variability and avoids inflating significance by incorrectly assuming that all measurements are independent.

Fixed effect estimates are reported with 95% confidence intervals (95% CI), which indicate the range within which the true effect is likely to fall, given the data and model. Confidence intervals were calculated using the *confint()* function, which provides an estimate of uncertainty for each model coefficient.

To evaluate model fit, we calculated both marginal R$^{2}$ and conditional R$^{2}$ using the *performance* package [21]. Marginal R$^{2}$ represents the proportion of variance explained by the fixed effects alone (temperature and water treatment), and shows how well the model performs due to the predictors of scientific interest. In contrast, conditional R$^{2}$ includes both fixed and random effects, and thus reflects the total variance explained by the model, including differences among rink sections. A large difference between marginal and conditional R$^{2}$ indicates substantial between-section variation, while a small gap suggests the fixed effects explain most of the observed pattern. To ensure the validity of our models, we verified that model assumptions were met. In particular, we assessed the normality and homoscedasticity of residuals through diagnostic plots and Shapiro-Wilk tests. The residuals from all models approximated a normal distribution, and no strong patterns were observed in residual vs. fitted plots. These checks confirm that linear mixed-effect models were appropriate for the data and that key assumptions of linearity and normally distributed errors were reasonably satisfied.

Models were specified without a global intercept so that treatment coefficients for 220 ppm, 80 ppm, and 5 ppm represent baseline values for each treatment. The fixed-effect model structure was:

$$\begin{aligned} \text {Hardness or coefficient of friction}&= \text {Treatment coefficient(s)} \\&\quad + \bigl (\text {Temperature coefficient} \times \text {Temperature}\bigr ) \\&\quad + \text {Section random intercept} \end{aligned}$$

This statistical framework provides a rigorous yet flexible approach, making use of replication while avoiding the need to rebuild the rink for each new treatment. It enabled meaningful comparisons across treatment conditions under realistic operating constraints.

## Results

**Table 1:** Water chemistry parameters for the three treatment types used in the experiment

Parameter	Unit	220 ppm	80 ppm	5 ppm
Total Dissolved Solids	mg/L	220 ± 11	80 ± 4	5 ± 0.3
Conductivity	$\mu$s/cm	240.6	112.7	6.9
pH	unitless	7.8	7.4	6.0
Water Hardness	mg/L as CaCO$_3$	123.7	46.4	3.8
Alkalinity	mg/L as CaCO$_3$	89.4	31.6	1.2
Inorganic Carbon	mg/L	22.3	8.4	1.0
Dissolved Organic Carbon	mg/L	1.7	0.65	0.35
Dissolved Organic Nitrogen	mg/L	0.14	0.12	0.08
Sodium	mg/L	13.8	5.4	0.8
Potassium	mg/L	1.7	0.8	0
Calcium	mg/L	34.7	13.0	0.4
Magnesium	mg/L	9.0	3.4	0.7
Lithium	mg/L	0.001	0	0.013
Ammonium	mg/L	0.50	0.32	0.21
Fluoride	mg/L	0.59	0.22	0.05
Chloride	mg/L	24.3	8.9	0.5
Nitrate	mg/L	1.3	0.5	0.2
Phosphate	mg/L	1.8	0.6	0
Sulfate	mg/L	21.6	7.7	0.5
Bicarbonate	mg/L	108.4	38.4	1.4
Carbonate	mg/L	0.3	0	0

The 220 ppm water represents untreated Toronto tap water, while the 80 ppm and 5 ppm waters were treated to achieve lower total dissolved solids (TDS) levels. Parameters include pH, conductivity, water hardness, alkalinity, inorganic and organic carbon, dissolved ions, and other chemical properties. Values for total dissolved solids are presented as the mean ±5% error, where the error term represents estimated relative uncertainty in calculated TDS (sum of measured constituents)

### Water chemistry

Water chemistry differed across treatments (Table 1). The 220 ppm TDS water had higher pH (7.8), hardness (123.7 mg/L as CaCO$_3$), and conductivity (240.6 $\mu$S/cm) than the 80 ppm and 5 ppm treatments. The 5 ppm treatment showed near-complete removal of major ions, with calcium and magnesium reduced to 0.4 mg/L and 0.7 mg/L, respectively. Dissolved organic carbon was 0.35 mg/L in the 5 ppm treatment.

### Ice hardness

**Fig. 2:**
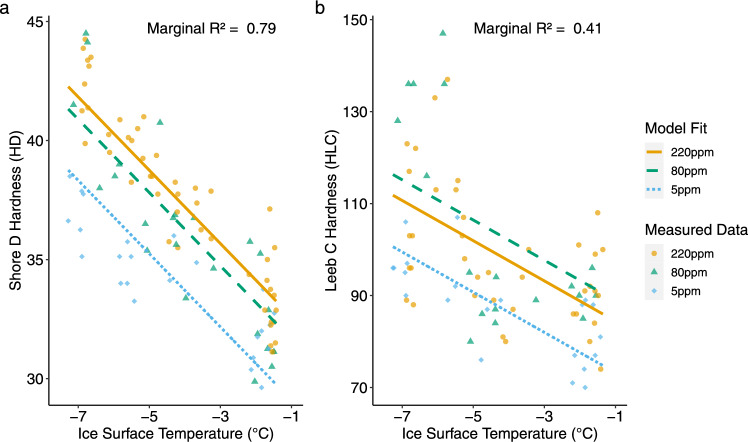
Hardness measurements and mixed-effect model fits for Shore D and Leeb C testers, showing the fixed effects of treatment and ice surface temperature. **a** Shore D hardness measurements (HD) with model fits for each treatment (220 ppm, 80 ppm, and 5 ppm total dissolved solids). The fixed effects in the Shore D model explain 79% of the variance (marginal R$^{2}$ = 0.79). **b** Leeb C hardness measurements (HLC) with model fits for each treatment. The fixed effects in the Leeb C model explain 41% of the variance (marginal R$^{2}$ = 0.41). Data points are colour-coded by treatment: 220 ppm in gold, 80 ppm in blue, and 5 ppm in green. Model fits are based on the fixed-effect coefficients presented in Table 2 and follow the form: Hardness = Treatment Coefficient + (Temperature Coefficient $\times$ Temperature in $^{\circ }$C)

**Table 2:** Mixed-effect model results for Shore D (HD), Leeb C (HLC), and Leeb D (HLD) hardness measurements

Model	Marginal R$^{2}$ (R$^{2}$m)	Conditional R$^{2}$ (R$^{2}$c)	Variable	Coefficient [95% CI]
Shore D	0.79	0.81	220 ppm	31.05 [30.10, 32.02]
			80 ppm	30.11 [29.05, 31.15]
			5 ppm	27.55 [26.46, 28.61]
			Ice Surface Temp	−1.54 [−1.70, −1.37]
Leeb C	0.41	0.41	220 ppm	80.06 [72.93, 87.28]
			80 ppm	84.55 [75.92, 93.03]
			5 ppm	68.87 [60.82, 76.97]
			Ice Surface Temp	−4.37 [−5.77, −2.97]
Leeb D	0.07	0.1	220 ppm	115.49 [106.94, 124.49]
			80 ppm	113.98 [105.31, 122.70]
			5 ppm	128.88 [118.36, 138.81]
			Ice Surface Temp	0.46 [−1.01, 1.98]

Coefficients are shown with their 95% confidence intervals [95% CI], which reflect the range within which the true value is likely to fall. Fixed effects include water treatment type (220 ppm, 80 ppm, and 5 ppm total dissolved solids) and ice surface temperature. Marginal R$^{2}$ (R$^{2}$m) represents the proportion of variance explained by fixed effects alone (treatment and temperature), while conditional R$^{2}$ (R$^{2}$c) includes both fixed and random effects (variation between rink sections). Leeb D results are included for transparency but showed weaker explanatory power, as reflected by the low R$^{2}$ values and overlapping 95% CI

Shore D hardness showed effects of water treatment and ice surface temperature (Table 2; Fig. 2a). Ice made with 220 ppm water had a treatment coefficient of 31.05 (95% CI [30.10, 32.02]), compared to 30.11 (95% CI [29.05, 31.15]) for 80 ppm and 27.55 (95% CI [26.46, 28.61]) for 5 ppm. Ice surface temperature had a negative effect, with a slope of $-1.54$ (95% CI [$-1.70$, $-1.37$]) Shore D units per $^\circ$C. Fixed effects explained 79% of the variance (marginal $R^2$ = 0.79) and the total variance explained was 81% (conditional $R^2$ = 0.81).

Leeb C hardness results were broadly consistent with Shore D measurements (Table  2 and Fig.  2b). Treatment coefficients for 220 ppm was 80.06 (95% CI [72.93, 87.28]), 84.55 (95% CI [75.92, 93.03]) for 80 ppm, and 68.87 (95% CI [60.82, 76.97]) for 5 ppm. Surface temperature decreased Leeb C hardness (HLC) by −4.37 (95% CI [−5.77, −2.97]) units per $^{\circ }$C. Both Shore D and Leeb C testers indicated that 5 ppm water produced the least hard ice, while 80 ppm and 220 ppm treatments resulted in similar hardness values with overlapping 95% CI. Fixed effects explained 41% of the variance (marginal R$^{2}$ = 0.41), indicating moderate predictive power relative to Shore D.

Leeb D hardness measurements were less reliable than both Shore D and Leeb C results (Table 2). Treatment coefficients were 115.49 (95% CI [106.94, 124.49]) for 220 ppm, 113.98 (95% CI [105.31, 122.70]) for 80 ppm, and 128.88 (95% CI [118.36, 138.81]) for 5 ppm. The temperature effect was 0.46 (95% CI [−1.01, 1.98]) units per $^{\circ }$C, crossing zero. Marginal R$^{2}$ = 0.07, indicating weak explanatory power. Given the wide CIs and low model fit, Leeb D results showed limited sensitivity to treatment and temperature under these conditions.

### Friction

**Fig. 3:**
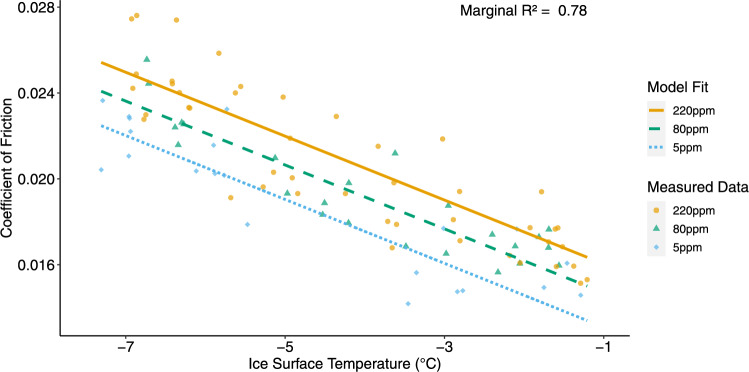
Skate-to-ice static friction measurements and mixed-effect model fits, showing the fixed effects of treatment and ice surface temperature. The coefficient of friction is plotted as a function of ice surface temperature for the three treatments: 220 ppm (gold), 80 ppm (blue), and 5 ppm (green). Points represent individual measurements, and lines represent the fits from the mixed-effect model. The fixed effects explain 78% of the variance (marginal R$^{2}$ = 0.78). Model fit is based on the fixed-effect coefficients presented in Table 3 and follows the form: Coefficient of Friction = Treatment Coefficient + (Temperature Coefficient $\times$ Temperature in $^{\circ }$C)

**Table 3:** Mixed-effect model results for static friction measurements

Model	Marginal R$^{2}$	Conditional R$^{2}$	Variable	Estimate [95% CI]
	(R$^{2}$m)	(R$^{2}$c)		
Friction	0.78	0.8	220 ppm	0.0146 [0.0135, 0.0155]
			80 ppm	0.0132 [0.0122, 0.0143]
			5 ppm	0.0116 [0.0105, 0.0129]
			Ice Surface Temp	−0.0015 [−0.00165, −0.00132]

Coefficients are reported with their 95% confidence intervals [95% CI], representing the likely range of the true effect. Fixed effects include water treatment type (220 ppm, 80 ppm, and 5 ppm total dissolved solids) and ice surface temperature. Marginal R$^{2}$ (R$^{2}$ $_m$) reflects the proportion of variance explained by the fixed effects alone, while conditional R$^{2}$ (R$^{2}$ $_c$) accounts for both fixed and random effects, including variation between rink sections

The coefficient of static friction varied significantly between water treatments (Table 3 and Fig. 3). Ice made with 220 ppm water had a treatment coefficient of 0.0146 (95% CI [0.0135, 0.0155]), compared to 0.0132 ([0.0122, 0.0143]) for 80 ppm and 0.0116 ([0.0105, 0.0129]) for 5 ppm. These results suggest that higher TDS levels increase the resistance between the skate blade and the ice surface. The fixed effects explained 78% of the variance in the friction model, indicating strong predictive power for treatment and temperature effects.

Ice surface temperature influenced friction, with a negative relationship observed across all treatments (Treatment Coefficient = −0.00149 [−0.00165, −0.00132] per $^{\circ }$C). As the temperature increased, the coefficient of friction decreased, reflecting a reduction in resistance to motion at warmer temperatures. The conditional R$^{2}$ was 80%, indicating that only 2% of the variance was due to random section-to-section differences not attributable to treatment or temperature.

## Discussion

In this study, we demonstrated that nondestructive testing methods can effectively assess ice hardness and skate-on-ice static friction in a controlled rink environment under varying water quality conditions. The Shore D indentation tester provided the most consistent and reliable measurements, followed by the Leeb C rebound tester. In contrast, the Leeb D tester showed poor model fit and was less suitable for rink ice assessment. Water quality influenced both hardness and friction, with the lowest TDS treatment (5 ppm) producing the softest ice and the least friction. Ice made with higher TDS levels (80 ppm and 220 ppm) was harder, though the differences between these treatments were small, as detected by both the Shore D and Leeb C testers. Temperature influenced both hardness and static friction, with warmer ice consistently softer and lower in friction, reinforcing the importance of precisely controlling surface temperature in rink management. These findings provide insight into how water quality and environmental conditions influence ice properties in realistic skating environments.

To be effective for ice rink testing, measurement methods must be nondestructive, accurate, and portable. The Shore D tester’s consistent performance across all treatments and temperatures makes it particularly well suited for practical rink applications. The Leeb C tester also showed promise for thinner ice layers; in dry ice tests, Kaufmann et al. [22] reported that Leeb C impacts produced fewer cracking events than Leeb D impacts. Given that indentation-based methods directly assess resistance to deformation, Shore D measurements may be more representative of the mechanical stresses imposed by skates than rebound-based methods. This interpretation is supported by Zhang et al. [6], who used Shore D hardness to evaluate ice quality in winter sports rinks. However, hardness alone does not fully capture how ice behaves under dynamic conditions. Friction at the skate-ice interface involves localized high-pressure zones and the formation of ice-rich slurries [13]. Our results demonstrate that a static friction index, measured using a custom apparatus with actual skate blades, is sensitive to both ice surface temperature and water treatment. While further research is needed to connect these measurements to sport-specific performance outcomes, the approach offers a practical and reproducible tool for comparing ice conditions in real-world rink environments.

### Water quality and ice hardness

Impurities in water, measured as TDS, can influence ice hardness, strength, and durability, although the mechanisms remain unclear. In this study, ice made with 5 ppm water was consistently softer (lower Shore D and Leeb C hardness) than ice made with 80 ppm and 220 ppm water across the tested temperature range. This pattern suggests that ultra-low TDS can reduce rink-ice hardness, whereas moderately low TDS may retain hardness comparable to typical municipal water. More broadly, the observed sensitivity of hardness to TDS at the lowest concentrations is consistent with the idea that water-treatment targets may differ among ice sports, reflecting differing ice–equipment interactions (e.g., skate–ice versus stone–ice contact).

Experimental studies have attempted to assess the role of TDS in ice properties, though prior investigations did not always isolate TDS as the primary variable. Zhang et al. [5] examined the relationship between TDS and ice mechanical properties but also evaluated freezing layer thickness and structural variations. Their findings indicated that increasing TDS from 10 to 100 ppm reduced compressive and elastic strength, suggesting that higher impurity levels produce softer, less durable ice. However, a subsequent study by Zhang et al. [6] found a more complex relationship, reporting that ice hardness was influenced not only by TDS but also by freezing conditions and ice formation processes.

A key limitation of these studies is the ice formation process itself. Both Zhang et al. articles [5, 6] froze their samples in 5 mm layers, which is thicker than the thin-layer methods used in standard rink operations. In contrast, professional rink ice is constructed by freezing the thinnest possible layers (typically less than 1 mm at a time); both Zhang et al. [5, 6] noted that the thinnest layer they tested (1 mm) produced stronger and more uniform ice. This supports the industry consensus that layering in minimal increments enhances structural integrity. Zhang et al. [5] also reported that thicker frozen layers contained more air bubbles, which can interfere with crystal growth and contribute to mechanical weakening.

This view is supported by foundational work from Michel and Ramseier [23], who showed that slower directional freezing in calm conditions promotes the formation of large, horizontally aligned tabular crystals with preferred c-axis orientations, which are associated with stronger and more uniform ice. In contrast, thicker or rapidly frozen layers tend to produce smaller, randomly oriented crystals and increased air entrapment, both of which reduce ice strength. Leppäranta [24] further emphasizes that temperature gradients and freezing rates play a key role in determining the mechanical properties of lake ice, supporting the rationale for thin-layer construction in professional rinks. These structural differences complicate interpretation of TDS effects, as porosity and crystal orientation may confound direct comparisons between water treatments.

Another important methodological difference is sample size and statistical robustness. Both Zhang et al. articles [5, 6] used only three measurements per group, potentially limiting their ability to detect subtle effects. In contrast, our study was designed to test the effect of TDS in a statistically robust manner. Each observation in our study represents a mean of eight measurements, and we had three independently treated rink sections for each water quality treatment in the experiments. This approach improves precision and reliability and provides repeated measures across a range of ice temperatures, increasing the statistical power to detect meaningful differences.

Furthermore, while Zhang et al. [5, 6] compared nondestructive hardness testing to destructive compressive strength tests, their studies were not designed to isolate TDS effects in an applied rink environment. In contrast, our study explicitly aimed to quantify the influence of TDS on ice hardness and friction under conditions that mirror operational ice rinks. This may explain why our results showed a clearer effect of TDS, as our experimental design had the statistical power to detect these relationships.

The precise mechanisms by which impurities influence ice hardness remain an open question. While it is generally assumed that dissolved solids are excluded from the ice lattice and accumulate at grain boundaries [25], recent work by Yashima et al. [26] suggests that certain ions, such as chloride and potassium, can be incorporated into the ice matrix. This could have implications for ice strength, affecting its response to temperature fluctuations and mechanical stress. Further studies are needed to determine whether specific ions within TDS contribute more to ice hardness than others and how freezing dynamics interact with water chemistry to influence ice quality.

Taken together, our findings reinforce that water quality is a key but not the sole determinant of ice hardness in rinks. While ultra-pure water (5 ppm TDS) produced softer ice, the differences between 80 ppm and 220 ppm were negligible with overlapping confidence intervals, suggesting that moderate impurity levels may be sufficient to maintain high-quality rink conditions. Future work should explore whether different ion compositions within TDS influence ice strength differently and examine how resurfacing techniques interact with water quality to affect long-term ice formation, mechanical strength, wear, and overall rink performance.

### Water quality and ice friction

Lower TDS levels reduced the static friction index, with reductions of similar magnitude observed from 220 ppm to 80 ppm and from 80 ppm to 5 ppm. The frictional reduction associated with lowering TDS was comparable in size to that produced by a 1$^\circ$C increase in ice surface temperature. This indicates that TDS influences skate-to-ice friction under rink conditions, potentially through changes in surface structure, melt-layer dynamics, or lubrication at the interface.

In hockey rink operations, TDS targets are often maintained in a moderately low range [8]. In our experiments, the 80 ppm treatment produced hardness comparable to 220 ppm while reducing the static friction index by an amount equivalent to warming the ice by about 1$^\circ$C. This suggests that moderately reduced TDS can lower skate-to-ice friction without substantially reducing hardness under the conditions tested.

Low TDS water (<10 ppm) is also used in curling [9], but friction mechanisms differ across applications (e.g., skate blade–ice contact versus stone–pebble contact on pebbled ice). Further work is needed to determine whether similar TDS effects occur for stone–ice friction on curling-specific surfaces.

Temperature effects are discussed below; however, these results indicate that adjusting TDS provides an additional lever for modifying skate-to-ice friction under operationally relevant conditions. Future work should examine whether specific ion compositions within TDS influence friction differently and how resurfacing practices interact with water quality over longer time scales.

### Temperature effects on ice hardness and friction

**Fig. 4:**
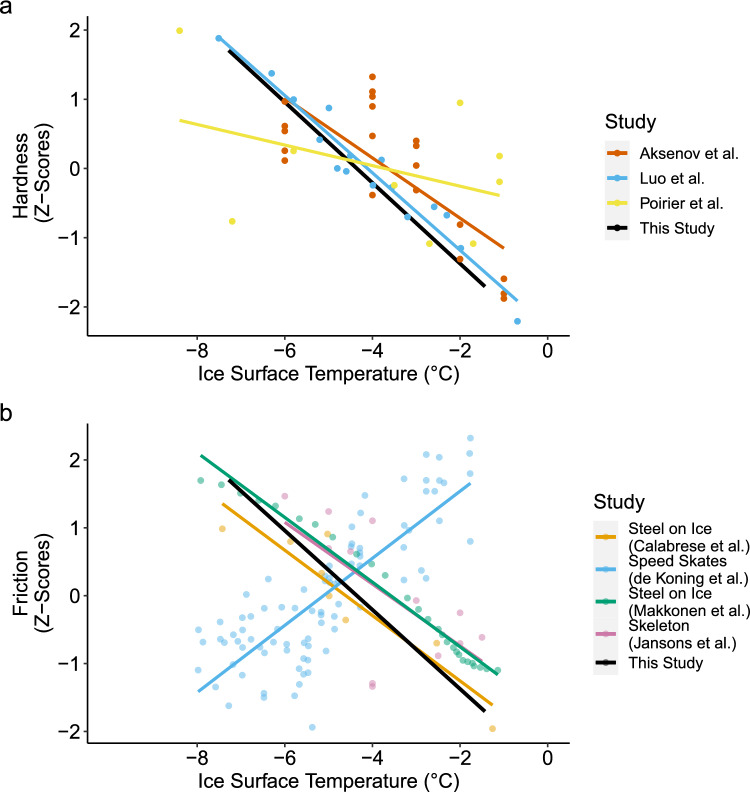
Comparison of hardness and friction with ice surface temperature across studies. **a** Hardness (z-scores) as a function of ice surface temperature with linear fits from Aksenov et al. [27], Luo et al. [28], and Poirier et al. [1], and the fixed effect of temperature from this study. **b** Friction (z-scores) as a function of ice surface temperature with linear fits from Calabrese et al. [16], de Koning et al. [2], Makkonen and Tikanmäki [29], Jansons et al. [30], and the fixed effect of temperature from this study. Hardness and friction values from each study were converted to z-scores (normalized to distance from the mean) within each study to allow for a comparison of patterns outside of experiment-specific conditions

Both the Shore D and Leeb C testers showed that ice hardness increased with decreasing temperature across all water treatments. This trend is consistent with the well-established relationship between ice temperature and hardness; colder ice is harder. Similarly, our skate-on-ice static friction measurements showed a consistent decrease in friction with increasing temperature across all treatments, indicating a reduction in resistance at the blade-ice interface under warmer conditions. These trends align with studies on ice hardness and ice friction systems, as shown in Fig. 4. To facilitate comparison across studies using different instruments and units, the datasets in Figure 4 were standardized using z-scores. This allows us to compare the relative sensitivity of each system to temperature while controlling for differences in absolute scale and measurement range. In terms of hardness (Fig. 4a), our results closely are similar to Aksenov et al. [27], Luo et al. [28], and Poirier et al. [1], all of which show increasing hardness as ice temperature decreases. The agreement across studies reinforces that the temperature dependence of hardness is a robust feature of ice mechanics, independent of experimental setup or measurement technique.

In Fig. 4b, we compare the isolated temperature effects from our static friction index with previously reported sliding resistance measurements and models across different steel-on-ice systems. Our static friction index, based on a controlled apparatus with skate blades pressed against the ice, is not equivalent to kinetic coefficients of kinetic friction, but it allows us to isolate how surface temperature alone influences resistance to motion. Over the narrow temperature range of our experiment (-2$^{\circ }$C to -7$^{\circ }$C), the relationship appears linear, with lower resistance at warmer temperatures. This trend aligns with tribometer-based measurements from Calabrese et al. [16] and with the modeling results of Makkonen and Tikanmäki [29], as well as with Jansons et al. [30], who used sliding time of a skeleton sled as a proxy for friction. However, this contrasts with the speed skating study by de Koning et al. [2], which reported increasing friction at higher ice temperatures. While our results support a linear trend across a narrow range, previous studies emphasize that friction on ice is shaped by complex multivariate interactions among ice temperature, sliding speed, normal force, surface roughness, and ambient humidity. The contrasting result from de Koning et al. merits examination to understand the conditions under which different temperature trends emerge.

The differences in blade geometry and contact mechanics between hockey skates and speed skates may help explain the contrasting temperature–friction trend observed in de Koning et al. [2]. In particular, plowing, where the blade cuts into softened ice and generates resistance, may be more pronounced at warmer temperatures and could contribute to the increased friction they reported. Our apparatus used hockey skate blades, which are rockered and curved along their length, likely reducing plowing resistance compared to long-track speed skating blades, which are flat and feature a nearly vertical leading edge. This almost 90-degree edge can penetrate more deeply into the ice as it softens, potentially increasing resistance at higher temperatures. In addition, the high speeds (8 m/s) tested by de Koning et al. may have introduced other mechanisms such as hydrodynamic lift, ice deformation, or aerodynamic drag, which are not relevant in our static setup. Long-track speed skating venues also tend to maintain colder ice surfaces than hockey rinks, possibly to mitigate such effects. Future work is needed to determine whether frictional responses differ fundamentally between hockey and speed skate blades across the range of temperatures used in ice arenas.

While our results suggest a linear relationship between temperature and our static friction index within the tested range, we acknowledge that this relationship may not hold across the full spectrum of ice temperatures. Du et al. [31] demonstrated that friction can increase sharply above –2 $^{\circ }$C due to deeper blade penetration and the formation of a thicker, more viscous melt layer. These higher temperatures lead to greater ice softening and lubrication effects that alter the dominant friction mechanisms. Although our study did not include ice temperatures above –2 $^{\circ }$C, which exceed the conditions typically maintained in arena settings, nonlinear behaviour likely emerges beyond this threshold. As such, our results represent only a narrow slice of a more complex, multivariate friction landscape involving temperature, velocity, pressure, humidity, and blade geometry.

Although static friction is not directly equivalent to kinetic friction, prior work suggests that both are governed by similar underlying mechanisms. In their foundational work on ice friction, Bowden and Tabor [14] demonstrated that static and kinetic friction show comparable trends with temperature in an ice-on-ice system, driven by the same processes of adhesion and deformation. In our case, the use of a static friction index provides a practical and repeatable proxy to isolate the effects of surface temperature and TDS under controlled conditions. Both static and kinetic friction on ice are known to be influenced by temperature, surface structure, and the presence of a liquid-like layer. Because they are governed by the same interfacial processes of adhesion and deformation, changes in static friction generally correspond to changes in kinetic friction, making it a useful indicator of how temperature and water quality influence ice conditions. Our approach allows for consistent comparisons across treatments without the confounding effects introduced by speed, momentum, or stride variability. While we present an index here rather than a direct measurement of kinetic friction, future work is needed to explicitly link this metric to sport-specific performance outcomes.

## Conclusion

Nondestructive measurements can quantify rink-ice hardness and a static skate-to-ice friction index under controlled conditions. Across treatments, ice made with 5 ppm TDS was softer and had a lower static friction index than ice made with 80 ppm and 220 ppm TDS, whereas 80 ppm and 220 ppm produced similar hardness with overlapping confidence intervals. Ice surface temperature affected both hardness and the friction index, with changes comparable in magnitude to those produced by the TDS reductions tested. Among the instruments evaluated, Shore D provided the clearest treatment and temperature responses, Leeb C showed similar trends with lower explanatory power, and Leeb D showed limited sensitivity under these conditions. These results are specific to the tested temperature range and to a static friction index; further work should evaluate dynamic, sport-specific friction and longer-term wear under operational resurfacing conditions.

## Supplementary Information

Below is the link to the electronic supplementary material.

Supplementary file 1 (pdf 146 KB)

## Data Availability

All data supporting the findings of this study are presented in the figures and can be extracted directly if needed. The underlying datasets are also available from the corresponding author upon reasonable request.
