# Supplementary material for: Effect of water quality on ice hardness and skate-to-ice friction in ice rinks
Source: Sports Eng. 2026 Jan 29;29(1):7. doi: 10.1007/s12283-025-00538-z (PMC12855353; doi:10.1007/s12283-025-00538-z)
Supplement: Supplementary file 1 — (pdf 146 KB) [file 12283_2025_538_MOESM1_ESM.pdf]

Supplementary Information for “Ice Hardness and Skate-to-Ice Friction in Ice Rinks across Varying Water Quality” in Sports Engineering

Ryan H. S. Hutchins[1]*, Jiani Wang[1], Stefania Impellizzeri[1*]

[1] Department of Chemistry and Biology, Toronto Metropolitan University, Toronto, ON M5B 2K3, Canada

* Corresponding Authors (email: ryan.hutchins@torontomu.ca; simpellizzeri@torontomu.ca)

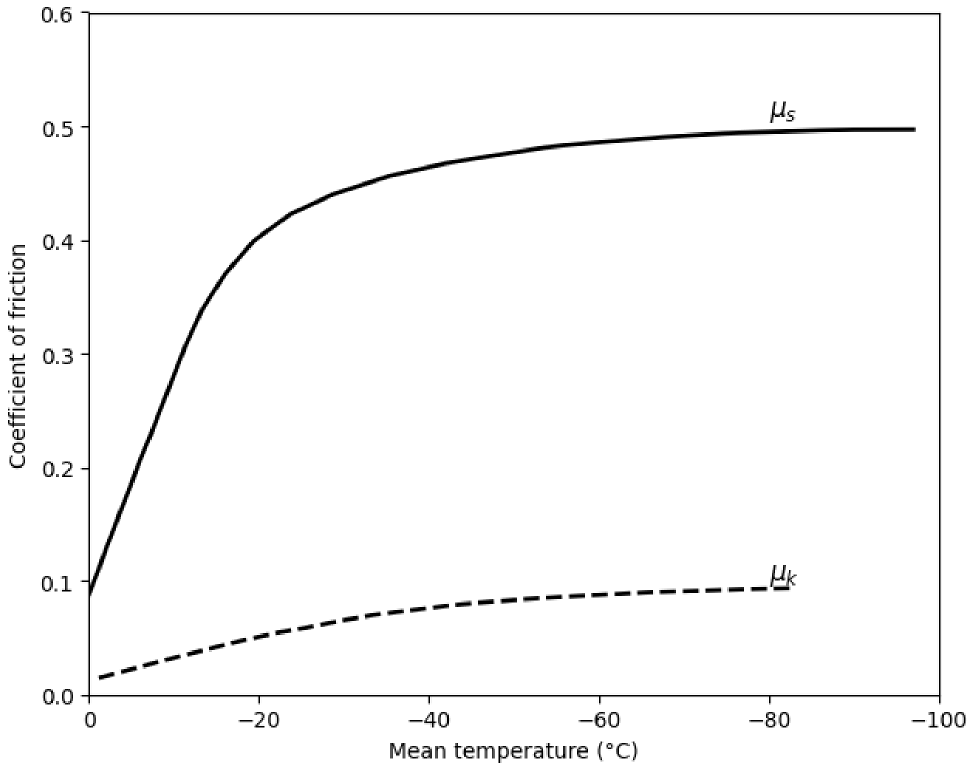

Figure S1. Static (μ_s) and kinetic (μ_k) friction coefficients versus mean ice temperature, replotted by the authors from digitized values corresponding to Fig. 27 in Bowden and Tabor[1].

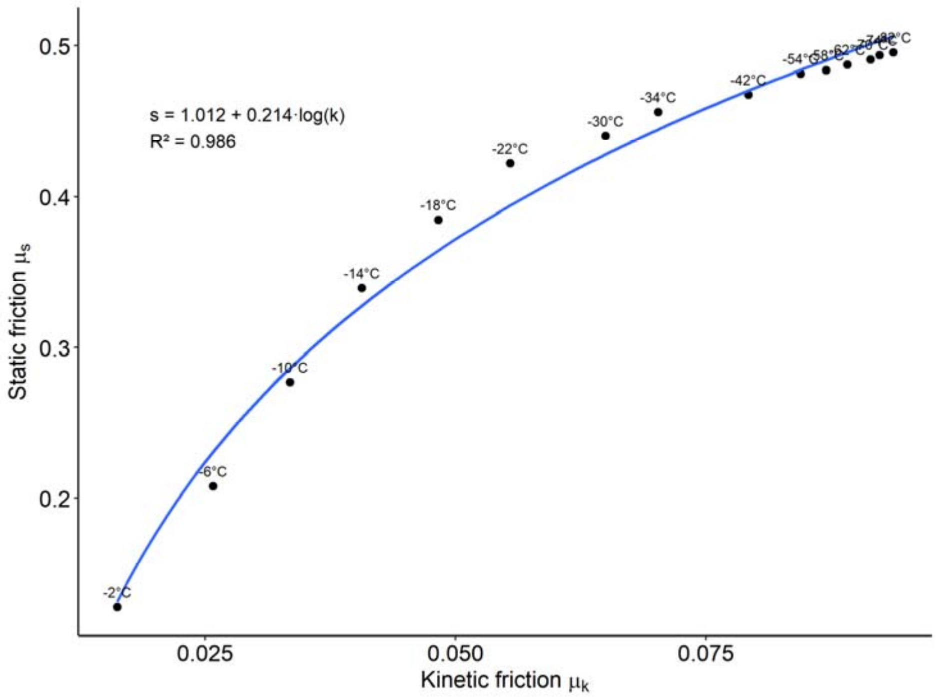

Figure S2. Relationship between static and kinetic friction based on the digitized data in Fig. S1. The log-linear model shows a linear relationship ($\mu_s = 1.012 + 0.214 \times \log(\mu_k)$, $R^2 = 0.986$), indicates that static and kinetic friction co-vary across temperature.

References

1. Bowden, F.P. & Tabor, D. The Friction and Lubrication of Solids. Oxford University Press, Oxford (2001). (Originally published 1950)
